# Theoretical Design of Novel Boron-Based Nanowires *via* Inverse Sandwich Clusters

**DOI:** 10.3389/fchem.2021.753617

**Published:** 2021-09-17

**Authors:** Cailian Jiang, Zhiwei Lv, Sudong Lv, Linwei Sai, Shukai Wang, Fengyu Li

**Affiliations:** ^1^ School of Physical Science and Technology, Inner Mongolia University, Hohhot, China; ^2^ College of Science, Hohai University, Changzhou, China

**Keywords:** first-principles, clusters, inverse sandwich structure, boron-based nanowires, magnetic and electronic properties

## Abstract

Borophene has important application value, boron nanomaterials doped with transition metal have wondrous structures and chemical bonding. However, little attention was paid to the boron nanowires (NWs). Inspired by the novel metal boron clusters Ln_2_B_*n*_^−^ (Ln = La, Pr, Tb, *n* = 7–9) adopting inverse sandwich configuration, we examined Sc_2_B_8_ and Y_2_B_8_ clusters in such novel structure and found that they are the global minima and show good stability. Thus, based on the novel structural moiety and first-principles calculations, we connected the inverse sandwich clusters into one-dimensional (1D) nanowires by sharing B−B bridges between adjacent clusters, and the 1D-Sc_4_B_24_ and 1D-Y_2_B_12_ were reached after structural relaxation. The two nanowires were identified to be stable in thermodynamical, dynamical and thermal aspects. Both nanowires are nonmagnetic, the 1D-Sc_4_B_24_ NW is a direct-bandgap semiconductor, while the 1D-Y_2_B_12_ NW shows metallic feature. Our theoretical results revealed that the inverse sandwich structure is the most energy-favored configuration for transition metal borides Sc_2_B_8_ and Y_2_B_8_, and the inverse sandwich motif can be extended to 1D nanowires, providing useful guidance for designing novel boron-based nanowires with diverse electronic properties.

## Introduction

Boron-based materials were found wide applications in the fileds of emissions, supercapacitors, optical absorptions, photodetectors, *etc.* (Xu et al., 2013; Sussardi et al., 2015; Akopov et al., 2017; Carenco et al., 2013; Tian et al., 2019)*.* Unlike the extensive attention on carbon clusters such as fullerenes and carbon fibers, boron clusters and materials are relatively less studied by scientists. However, there is much space and potential to develop boron-based nanomaterials.

Boron shows a strong tendency to form multi-center-two-electron bonds (mc-2e) in both polyhedral molecules and bulk isotopes (Wang, 2016; Jian et al., 2019; Lipscomb, 1977; Alexandrova et al., 2006) due to its electron deficiency. Therefore, boron clusters have the characteristic of electron delocalization bonding with some delocalized electronic structures and unique aromaticity (Li et al., 2018). In the past two decades, the structure and chemical bonding of bare boron clusters have been studied by combining experimental and theoretical methods (Li et al., 2017; Li et al., 2017; Pan et al., 2019), and planar clusters, nanotube-like cluster structures, graphene-like boron spheres and fullerene-like boron spheres have been found (Kiran et al., 2005; Piazza et al., 2014; Li et al., 2014; Bai et al., 2019; Zhai et al., 2014). Also due to the characteristic of electron deficiency, boron can be doped with metal to form different kinds of metal boride structures. Boron has formed a large number of important boride materials, ranging from superconducting MgB_2_ and superhard transition metal borides to borides with extremely high thermal conductivity (Nagamatsu et al., 2001; Chung et al., 2007).

As the 5th element adjacent to carbon in the periodic table, ring and cage boron clusters have poor stability due to their electron-deficient properties. However, the introduction of transition metals can greatly improve the stability of boron clusters. Transition-metal-doped boron clusters have led to a new direction of boron nanomaterials, such as the metal-centered aromatic borometallic wheels and tubular metal-centered drums (Romanescu et al., 2011; Popov et al., 2015; Jian et al., 2016; Jian et al., 2016; Li et al., 2017). On the other hand, assembling boron clusters by doping them with different types of atoms is a potential way to change properties. For example, CoB_18_^‒^ and RhB_18_^‒^ planar clusters have been found, which makes it possible to dope metal with borographene (Li et al., 2016; Jian et al., 2016). Wang and Boldyrev’s joint research group have reported a variety of neutral or charged planar wheel clusters centered on supercoordination transition metals M©B_*n*_ (M = Fe, Co, Nb, Ru, Rh, Ir, Ta; *n* = 8–10) (Romanescu et al., 2011).

Recently, Wang’s experimental group and Li’s theoretical group jointly observed several new metal boron clusters Ln_2_B_*n*_^−^ (Ln = La, Pr, Tb; *n* = 7–9) with an inverse sandwich structure (Li et al., 2018; Chen et al., 2019). It is found that these clusters have the double aromatic properties of *π* and *σ* bonding contributions, showing high stability and symmetry, and the magnetization of B_8_^‒^ ring is high. The study provides a novel pattern for the design of new lanthanide borides, and a few inverse sandwich complexes were proposed (Wang et al., 2019; Cui et al., 2020; Shakerzadeh et al., 2020; Xiao et al., 2021). A few questions arise naturally: Would the transition metal borides adopt the inverse sandwich structure in a stable manner? Can the inverse sandwich structure motif be extended to periodic nanomaterials, like designing the super stable 1D-P_10_ nanowire and 2D-P_8_N_2_ nanosheet based on all pentagon containing P_8_ clusters (Wang et al., 2020; Dong et al., 2021)? Thus, in this work, by means of first-principles calculations, we examined the stability of M_2_B_8_ (M = Sc and Y) clusters with the inverse sandwich structure, and extended the inverse sandwich moiety to design novel boron-based nanowires (NWs). The constructed 1D-Sc_4_B_24_ and 1D-Y_2_B_12_ NWs show good stability, and the former/later one is a semiconductor/metal. Our theoretical work successfully extended the inverse sandwich moiety to the 1D crystals, which is helpful to design novel boron-based nanowires with diverse electronic properties.

## Methods

The comprehensive genetic algorithm (CGA) (Zhao et al., 2016) combined with the DMol^3^ program (Delley, 1990; Delley, 2000) was used to search the global minimum of Sc_2_B_8_ and Y_2_B_8_ clusters. The low-energy clusters generated by CGA were further optimized using density functional theory (DFT) implemented in the Vienna *Ab initio* Simulation Package (VASP) code (Kresse and Furthmuller, 1996; Kresse and Hafner, 1993; Kresse and Hafner, 1994). The exchange and correlation functional are defined by the generalized gradient approximation (GGA) with the Perdew–Burke–Ernzerhof (PBE) functional (Perdew et al., 1996). The k points of the geometric optimization and the molecular dynamics simulation were set to 1 × 7 × 1 and 1 × 3 × 1. The phonon spectra were calculated by VASP and Phonopy codes (Togo and Tanaka, 2015). Thermal stability was assessed at 300 and 500 K based on first-principles molecular dynamics [FPMD simulations conducted at the DFT level using a canonical ensemble having a constant number of atoms, volume with the temperature controlled by the Nosé-Hoover thermostat (Martyna et al., 1992; Kresse and Hafner, 1993)], and temperature (NVT) with 1 fs time steps for a total simulated time duration of 5 ps. The band structures of the designed nanowires were calculated by PBE and Heyd-Suseria-Ernzerhof (HSE06) hybrid functional (Heyd et al., 2003). To predict the clusters and nanowires in a more reliable manner, we also considered the PBE + D2 approach (Bučko et al., 2010). Almost no difference was found between the PBE-D2 and PBE structures and cohesive energies.

## Results

### Structure, Stability and Magnetic Properties of Sc_2_B_8_ and Y_2_B_8_ Clusters

**FIGURE 1:**
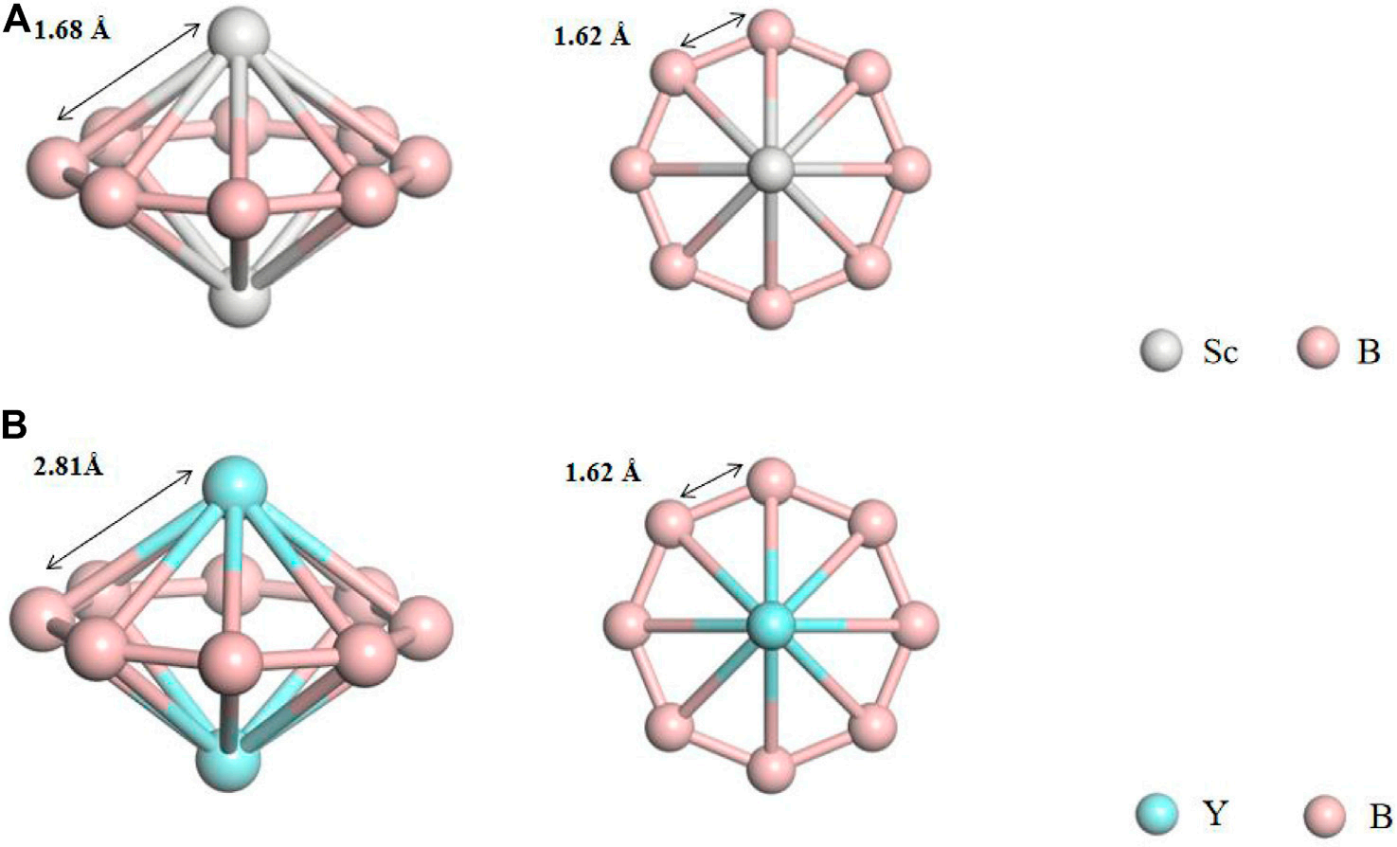
Side and top views of the optimized Sc_2_B_8_ **(A)** and Y_2_B_8_ **(B)** clusters.

Based on the inverse sandwich structure of La_2_B_8_^−^, we optimized the neutral transition metal boron clusters of the same configuration—Sc_2_B_8_ and Y_2_B_8_ clusters (the two Sc/Y atoms locate symmetrically to the two sides of the B_8_ ring). In Figure 1, M−B (M = Sc and Y) and B−B bond lengths in two cluster structures are given. For the cluster Sc_2_B_8_, the bond lengths of Sc−B (*d*_Sc−B_) and B−B (*d*_B−B_) are 1.68 and 1.62 Å, respectively. For the cluster Y_2_B_8_, Y−B bond length (*d*_Y−B_) is 2.81 Å and the bond length of B−B (*d*_B−B_) is 1.62 Å. Both two optimized neutral clusters well preserve the inverse sandwich structure of *D*_*8h*_ symmetry.

As shown in Supplementary Figure S1, the two vibrational spectra have simple vibration modes due to the high symmetry, and no negative mode was found, indicating the stability of these two clusters. In the Sc_2_B_8_ cluster, the intensity peaks of 144 and 752 cm^−1^ can be assigned to Sc−B bond and B−B bond vibrations, respectively. The sharp asymmetric oscillations in the Y_2_B_8_ cluster are at 149 and 721 cm^−1^, indicating the vibration modes of the Y−B bond and the B−B bond, respectively.

At the same time, a FPMD simulation lasting for 5 ps was performed for both clusters at room temperature (300 K). The annealed structures well remain the original inverse sandwich configuration, as shown in Supplementary Figure S2, which also suggests the good stability of the Sc_2_B_8_ and Y_2_B_8_ clusters adopting inverse sandwich structure.

Furthermore, CGA was used to generate low-energy isomers of Sc_2_B_8_ and Y_2_B_8_ clusters. The four low-lying structures, and an isomer, which can be viewed as the B-centered B_7_ ring sandwiched by two Sc/Y atoms, were presented in Supplementary Figure S3, and the inverse sandwich configuration for both Sc_2_B_8_ and Y_2_B_8_ clusters is the most stable one (0.69–1.34 eV lower than the other four low-energy isomers at PBE-D2 level of theory). In particular, the CCSD(T) test computations also support the PBE-D2 results that the inverse sandwich structures are much lower in energy than other isomers. Thus it is feasible to synthesize the inverse sandwich Sc_2_B_8_ and Y_2_B_8_ clusters in experiments.

Additionally, we examined the dissociation of inverse sandwich M_2_B_8_ (M = Sc, Y) clusters. For the first M dissociation (M_2_B_8_ → M + MB_8_), the reaction is endothermic by 2.11 and 2.08 eV, respectively for M = Sc and Y; and for removing the second M (MB_8_ → M + B_8_), it is also an endothermic reaction with the energy input of 2.37 and 2.17 eV for M = Sc and Y, respectively. The highly endothermic dissociations of M from B_8_, indicate reaction barriers are >2 eV. Meanwhile, when the M atoms were put 5 Å from the B_8_ center, it will be optimized to the energetically favored inverse sandwich structure. The above results as summarized in Supplementary Figure S4 again confirmed that the M_2_B_8_ (M = Sc, Y) clusters with inverse sandwich configuration are highly stable.

Besides, we further explored magnetic properties of the global minimum structures. Three magnetic configurations were compared, namely, antiferromagnetic (AFM), ferromagnetic (FM) and nonmagnetic (NM) states. We set the energy value of NM as 0 eV and all other energy values as their relative differences. Our calculations revealed that both Sc_2_B_8_ and Y_2_B_8_ clusters are nonmagnetic (Table 1).

**TABLE 1:** Relative energies of Sc_2_B_8_ and Y_2_B_8_ clusters with different magnetic configurations (in eV).

	NM	FM	AFM
**Sc_2_B_8_**	0.00	0.00	0.00
**Y_2_B_8_**	0.00	0.00	0.00

### Structure and Stability of 1D Nanowires

**FIGURE 2:**
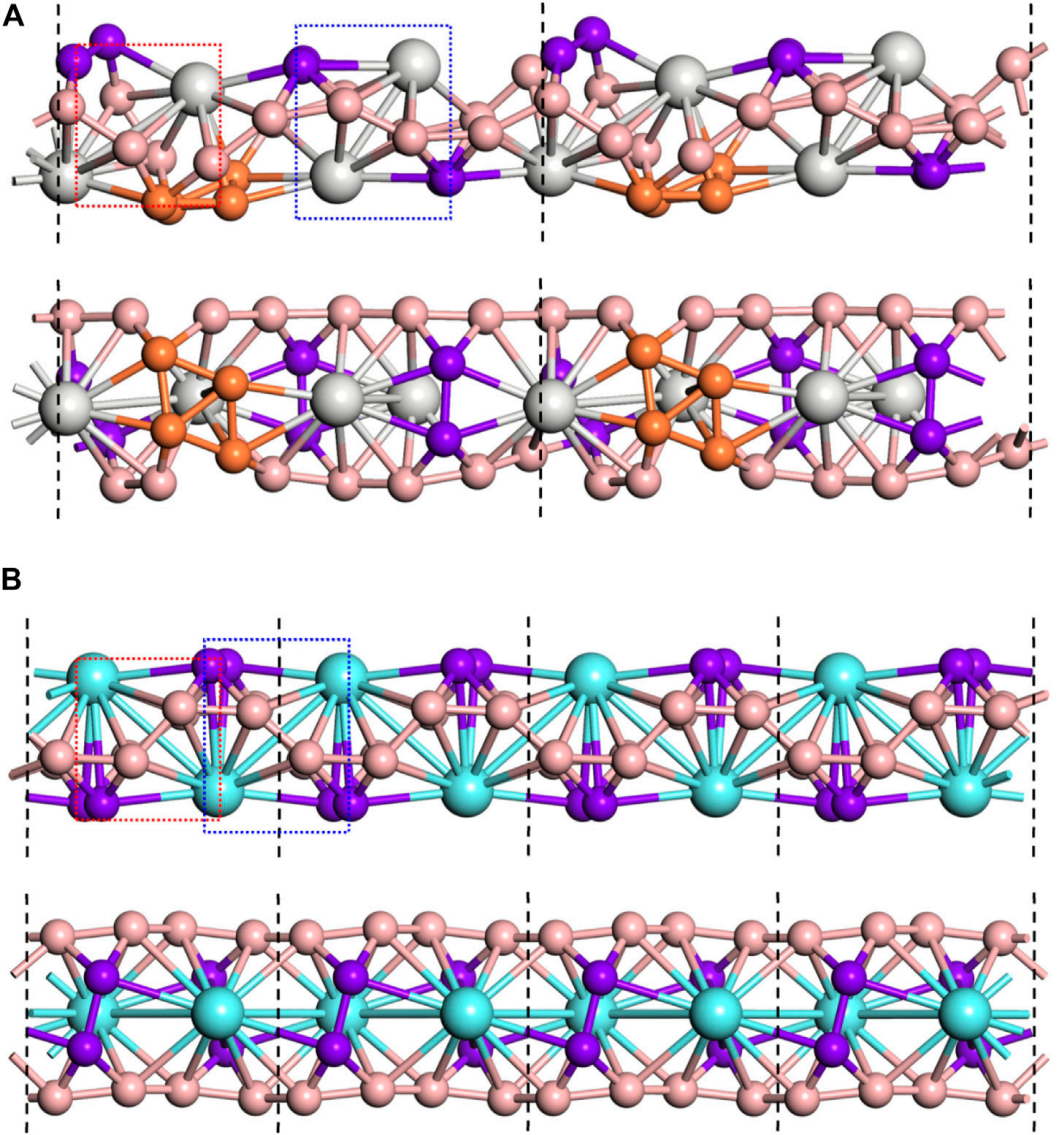
Two views of the 1D-Sc_4_B_24_ NW **(A)** and 1D-Y_2_B_12_ NW **(B)**. The unitcell was marked by black dashed lines. The inverse sandwich unit M_2_B_8_ was marked by red and blue dashed rectangle. The sharing B‒B bonds and the B rhombus were highlighted in purple and orange, respectively.

Considering that the Sc_2_B_8_ and Y_2_B_8_ clusters of inverse sandwich configuration are the global minima, the inverse sandwich structural moiety might be extended to a periodic manner. Therefore, we connected the inverse sandwich clusters into 1D nanowires by sharing B−B bridges between adjacent clusters, similar to the observation of inverse triple-decker La_3_B_14_^−^ (Chen et al., 2019). The 1D-Sc_4_B_24_ and 1D-Y_2_B_12_ nanowires were obtained after structural relaxation as displayed in Figure 2. For the optimized 1D-Sc_4_B_24_ (Figure 2A), neither the inverse sandwich moiety of Sc_2_B_8_ nor the sharing B−B bonds was clearly observed, largely due to the formation of B_4_ rhombus, which is regarded as a stable unit of boron analogs. The shared B–B (*d*_B−B_) key length is ∼1.59 Å, and the other B–B (*d*_B−B_) lengths are in the range of 1.58–1.62 Å. The Sc–B bond lengths (*d*_Sc−B_) are 2.41–2.49 Å. In contrast, for the 1D-Y_2_B_12_ NW (Figure 2B), the unitcell is formed by two Y_2_B_8_ clusters of inverse sandwich moiety by sharing a B−B bond. The length of the shared B−B bond (*d*_B−B_) is 1.56 Å, the lengths of others B−B bonds are ranged from 1.56 to 1.60 Å. The Y–B bond lengths (*d*_Y−B_) are ranged in 2.56–2.72 Å. Compared to the free cluster structures, the *d*_Y−B_ were compressed in 1D-Y_2_B_12_ nanowire, while the *d*_Sc−B_ were significantly stretched in the 1D-Sc_4_B_24_, indicating that although Sc_2_B_8_ and Y_2_B_8_ clusters have the same structure, they have different structural characteristics when forming one-dimensional nanowires.

In order to confirm the stability of the two nanowires, we first examined their thermodynamic stability by calculating the cohesive energy (*E*_*coh*_). In our work, the cohesive energy is defined as equation 1, where, *E*_*1*_/*E*_*2*_ is the energy of an isolated transition metal atoms (Sc or Y)/B atom, *E*_*tot*_ is the total energy of nanowire, *n*/*m* is the number of transition metal/B atoms.

According to the above definition of cohesive energy, the larger the calculated value is, the more stable the structure is. The calculated cohesive energies of 1D-Sc_4_B_24_ and 1D-Y_2_B_12_ nanowires are 5.92 and 6.00 eV/atom, respectively, much larger than the *E*_*coh*_ values of the clusters (5.35 and 5.29 eV/atom, respectively for Sc_2_B_8_ and Y_2_B_8_). These high cohesion energies show that two 1D nanowires have good thermodynamic stability.

**FIGURE 3:**
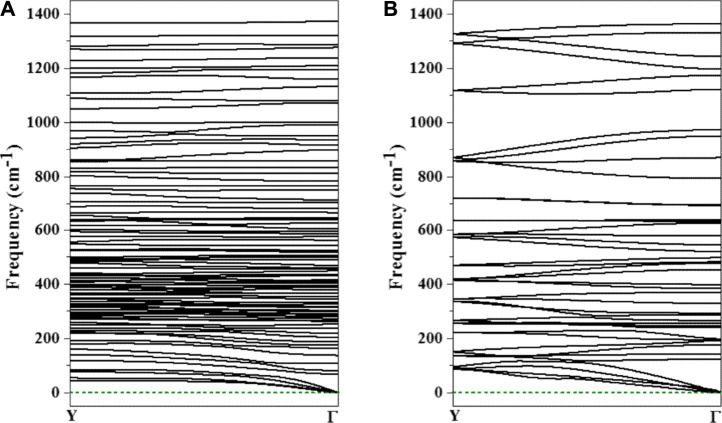
The calculated phonon spectra of the designed 1D-Sc_4_B_24_ **(A)** and 1D-Y_2_ B_12_ **(B)**.

Then, we calculated the phonon dispersion to investigate their dynamic stability. In these phonon dispersions, no imaginary frequencies were observed (Figure 3), indicating that the two designed nanowires based on the inverse sandwich Sc_2_B_8_ and Y_2_B_8_ clusters are dynamically stable.

Finally, we performed FPMD simulations in order to access their thermal stability with the supercell of 112 atoms (16 transition metal atoms and 96 B atoms). The 1D-Sc_16_B_96_ was annealed at 300 K for 5 ps, and the final structure retained the original B_8_ rings (Supplementary Figure S5A), and the structure obtained remains intact. For the one-dimensional nanowire structure constructed by Y_2_B_8_, we conducted two 5 ps simulation at room temperature of 300 K (Supplementary Figure S5B) and 500 K (Supplementary Figure S5), respectively. The 1D-Y_16_B_96_ structure still showed structural integrity under both simulation conditions. It also preserves structural integrity at 500 K in particular. The results of FPMD simulations confirm that two designed nanowires possess good thermal stability.

### Magnetic and Electronic Properties

**FIGURE 4:**
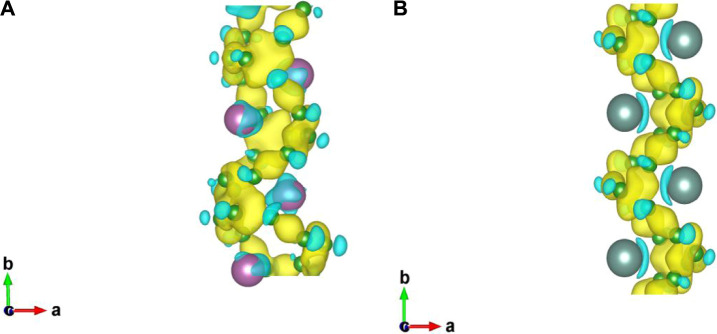
Differential charge density diagrams of designed nanowires 1D-Sc_4_B_24_ **(A)** and 1D-Y_2_B_12_ **(B)**. The isosurface value was set to be 0.015 e/Bohr^3^.

Through the above analysis of thermodynamic, dynamic and thermal stability, it is found that the two designed nanowires are stable. Therefore, we further explored the magnetic and electronic properties of the two nanowires. For the magnetic feature, five magnetic orderings were considered, namely AFM (including AFM1: − + − +, AFM2: + − − +, and AFM3: − − + +, Supplementary Figure S7, FM, and NM. Our computations showed that neither 1D-Sc_4_B_24_ nor 1D-Y_2_B_12_ is magnetic. The relative energies of examined magnetic configurations of the two structures were given in Table 2. In addition, through the analysis of charge transfer, we found that each Sc/Y atom transferred ∼1.5/2.0 electrons to boron. The differential charge density diagrams of the two 2D nanostructures (Figure 4) showed that the electrons have delocalized bonding characteristics.

**TABLE 2:** Relative energies of 1D- Sc_4_B_24_ and 1D-Y_2_B_12_ nanowires with various magnetic configurations (in eV).

	NM	FM	AFM1	AFM2	AFM3
**1D-Sc_4_B_24_**	0.00	0.00	0.00	0.00	0.00
**1D- Y_2_B_12_**	0.00	0.00	0.00	0.00	0.00

**FIGURE 5:**
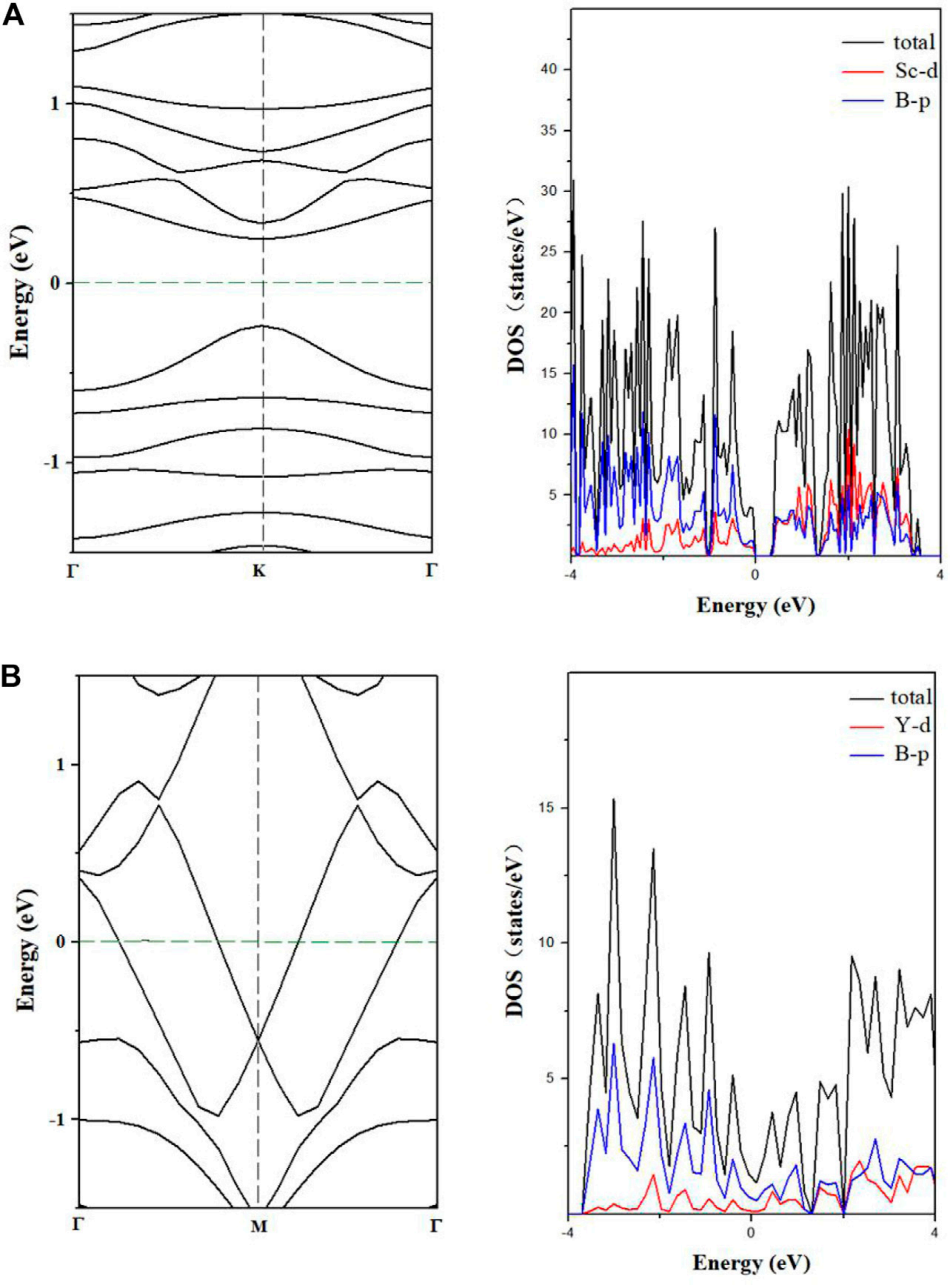
Energy band and density of states of 1D-Sc_4_B_24_ **(A)**, 1D-Y_2_B_12_ **(B)** nanowires predicted by PBE.

We used the PBE method to predict the electronic band structures of the two designed nanowires (Figure 5). Compared to the metallicity of teetotum cluster Li_2_FeB_14_ based nanowire (Shakerzadeh et al., 2020), the 1D-Sc_4_B_24_ nanowire is a direct-bandgap semiconductor with the bandgap of 0.51 eV, while the 1D-Y_2_B_12_ NW is a metal, and the p orbital of B dominates the state near the Fermi level. The commonly used PBE method usually underestimates the bandgaps. Therefore, we also used HSE06 method to calculate the electronic band structure of 1D-Sc_4_B_24_ nanowire, as shown in Supplementary Figure S8. The bandgap calculated by the HSE06 method is about 0.85 eV, 0.34 eV larger than the PBE value. The different electronic behavior of the two designed nanowires may originate from the different structures (Zeng et al., 2019).

## Conclusion

In summary, by means of first-principles calculations combined with CGA search, we found that Sc_2_B_8_ and Y_2_B_8_ clusters of inverse sandwich structure are the lowest-energy isomers and have good stability, and we constructed one-dimensional nanowires containing the structural moiety of the two clusters. The high stability of 1D-Sc_4_B_24_ and 1D-Y_2_B_12_ nanowires is confirmed by the investigation of thermodynamical, dynamical and thermal perspectives. Both 1D-Sc_4_B_24_ and 1D-Y_2_B_12_ nanowires are nonmagnetic; in terms of electronic behavior, the 1D-Sc_4_B_24_ is semiconducting with the HSE06 bandgap of 0.85 eV, while the 1D-Y_2_B_12_ is metallic. Our theoretical work not only identified the inverse sandwich configuration as the lowest-energy one for transition metal borides Sc_2_B_8_ and Y_2_B_8_ clusters, but also successfully extended the inverse sandwich moiety to 1D nanomaterials. Thus, it is helpful to design novel boron-based nanowires for both experimental and theoretical communities.

$$E_{coh}=\left(nE_{1}+mE_{2}-E_{tot}\right)/\left(n+m\right)$$

### Permission to Reuse and Copyright

Figures, tables, and images will be published under a Creative Commons CC-BY licence and permission must be obtained for use of copyrighted material from other sources (including re-published/adapted/modified/partial figures and images from the internet). It is the responsibility of the authors to acquire the licenses, to follow any citation instructions requested by third-party rights holders, and cover any supplementary charges.

## Data Availability

The original contributions presented in the study are included in the article/Supplementary Material, further inquiries can be directed to the corresponding authors.
