# Supplementary material for: Theoretical Design of Novel Boron-Based Nanowires via Inverse Sandwich Clusters
Source: Front Chem. 2021 Sep 17;9:753617. doi: 10.3389/fchem.2021.753617 (PMC8484529; doi:10.3389/fchem.2021.753617)
Supplement: Supplementary file 1 [file DataSheet1.docx]

Supplementary Material

# Supplementary Data

Supplementary Material should be uploaded separately on submission. Please include any supplementary data, figures and/or tables. All supplementary files are deposited to FigShare for permanent storage and receive a DOI.

Supplementary material is not typeset so please ensure that all information is clearly presented, the appropriate caption is included in the file and not in the manuscript, and that the style conforms to the rest of the article. To avoid discrepancies between the published article and the supplementary material, please do not add the title, author list, affiliations or correspondence in the supplementary files.

# Supplementary Figures and Tables

## Supplementary Figures

**(b)**

**(a)**

^
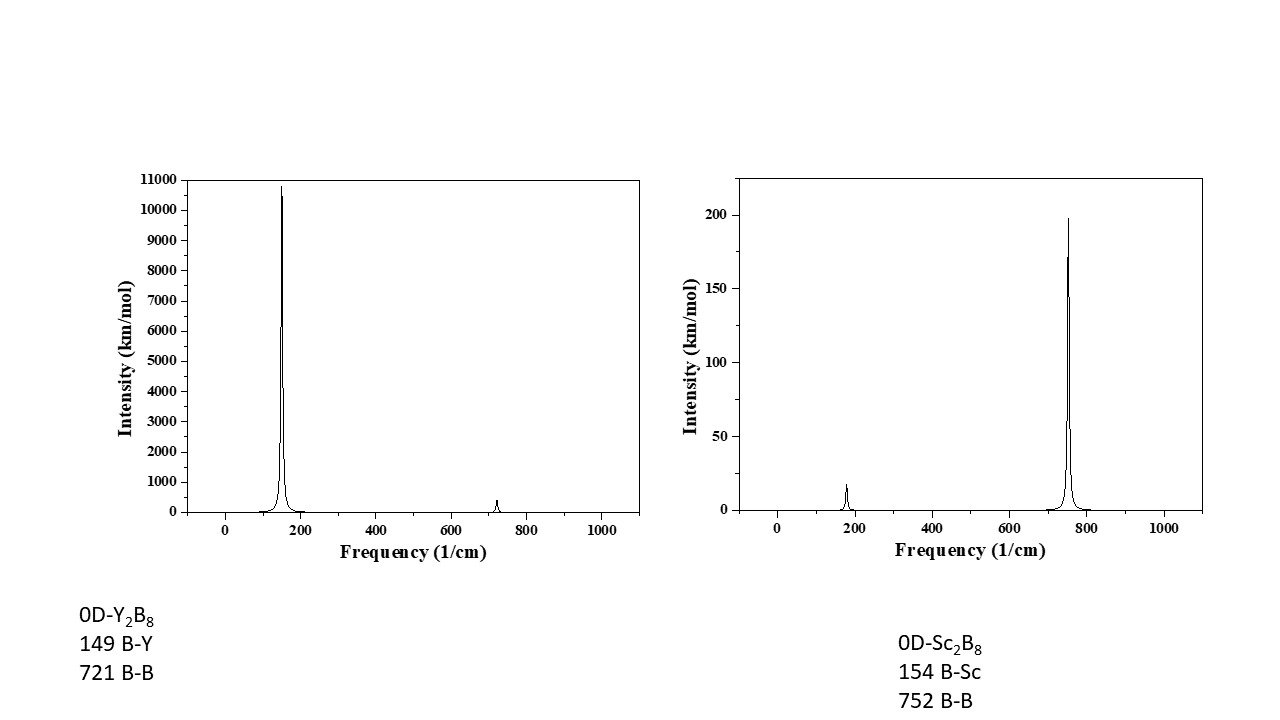
^

**FIGURE S1** Calculated frequency spectra of Sc_2_B_8_ (a) and Y_2_B_8_ (b) clusters


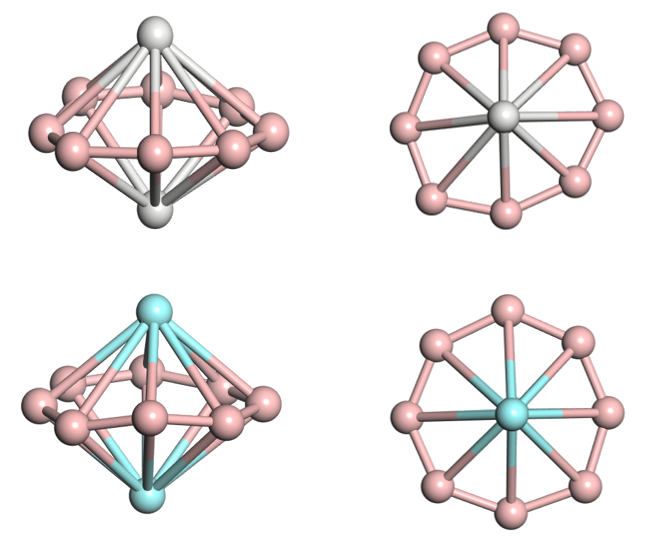


**(a)**
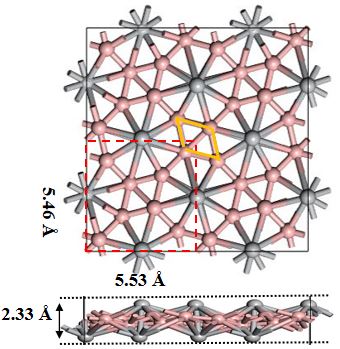

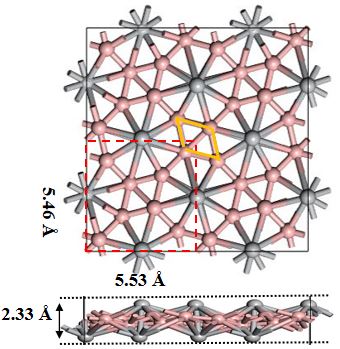


**(b))**
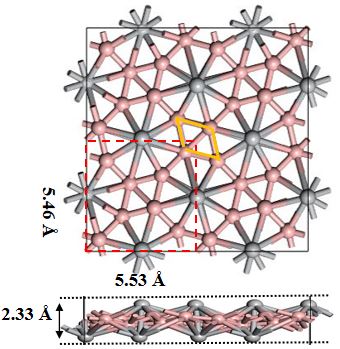

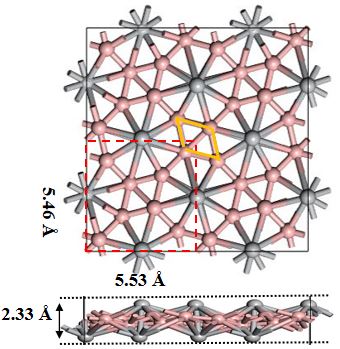


**FIGURE S2** The Sc_2_B_8_ (a) and Y_2_B_8_ (b) structural snapshot of the MD simulation at 300 K for at the end of 5 ps.


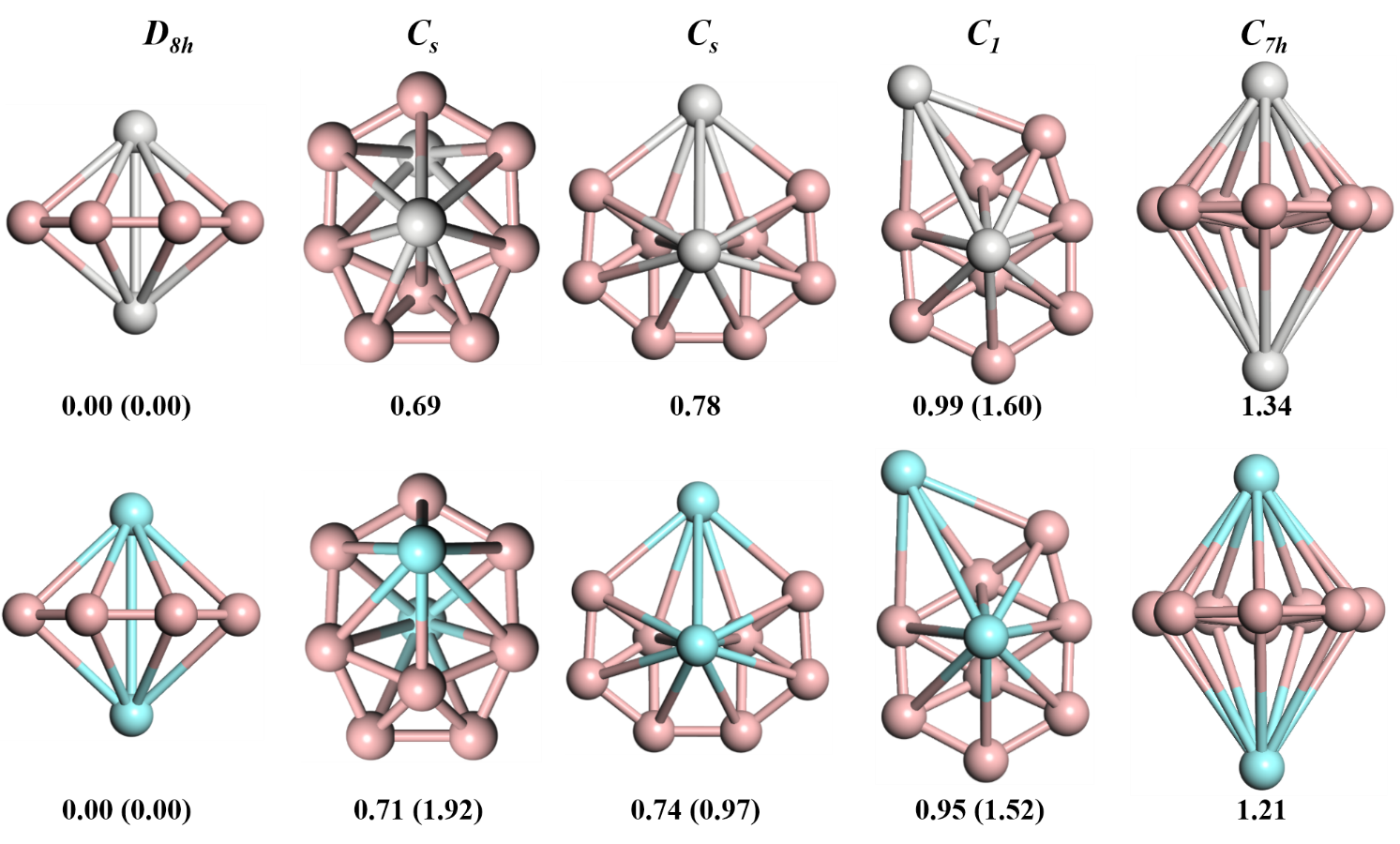


**FIGURE S3** The five low-lying structures of Sc_2_B_8_ (top panel) and Y_2_B_8_ (bottom panel) clusters generated by CGA. The relative energies (in eV computed at PBE-D2 level of theory) were given below the structures, data in parenthesis were computed at CCSD(T)/6-31G*~CEP31G level of theory based on the PBE+D2 optimized geometry using GAISSIAN 09 program [Frisch, M., Trucks, G., Schlegel, H., Scuseria, G., Robb, M., Cheeseman, J., et al. (2013). Gaussian 09, Revision E.01. Wallingford, CT: Gaussian Inc.].


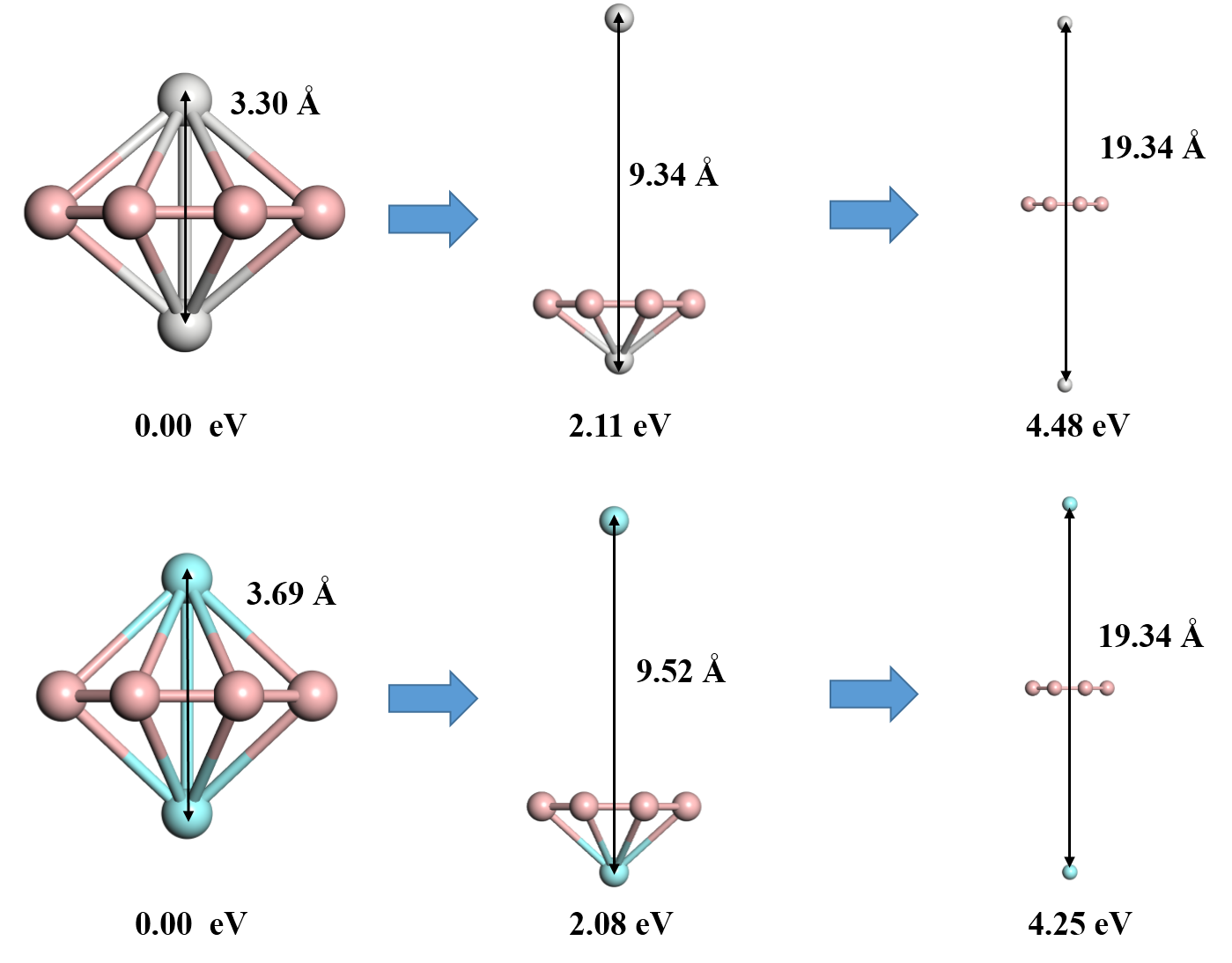


**FIGURE S4** The dissociations of Sc_2_B_8_ (a) and Y_2_B_8_ (b). The relative energies were given.


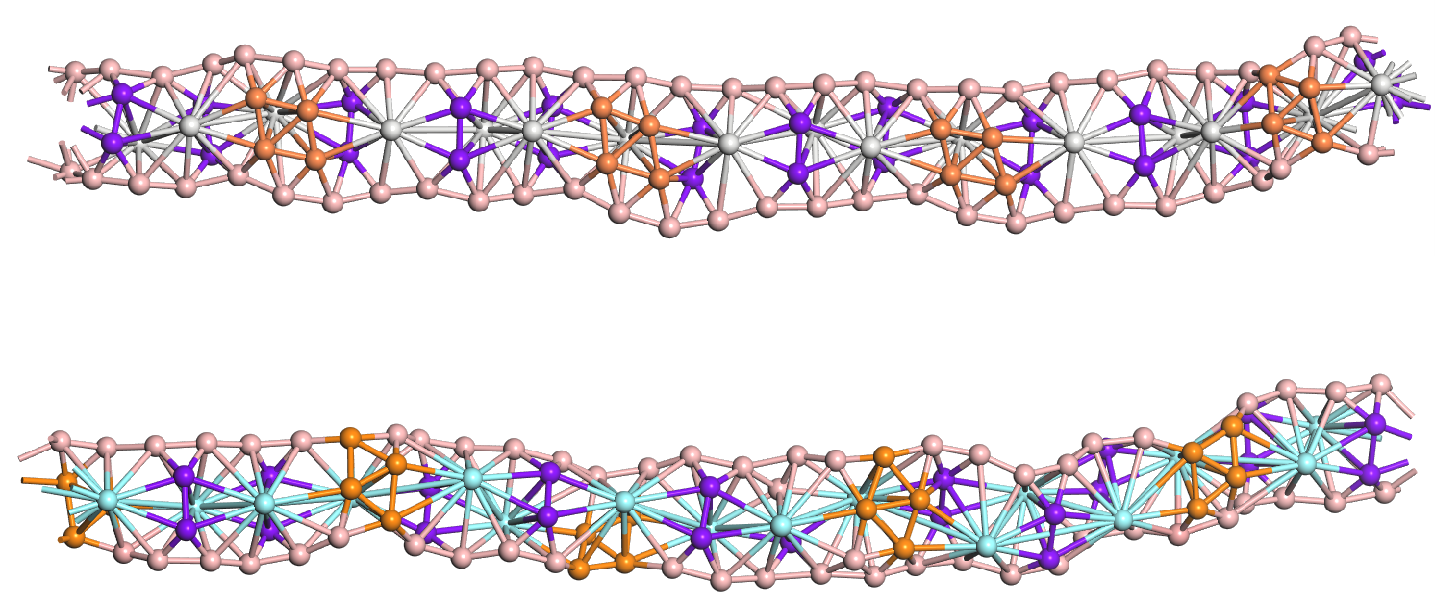


**(b)**

**(a)**

**FIGURE S5** Structure diagrams of 1D-Sc_4_B_24_ (a) and 1D-Y_2_B_12_ (b) after 5 ps’s FPMD simulation at 300 K.

**
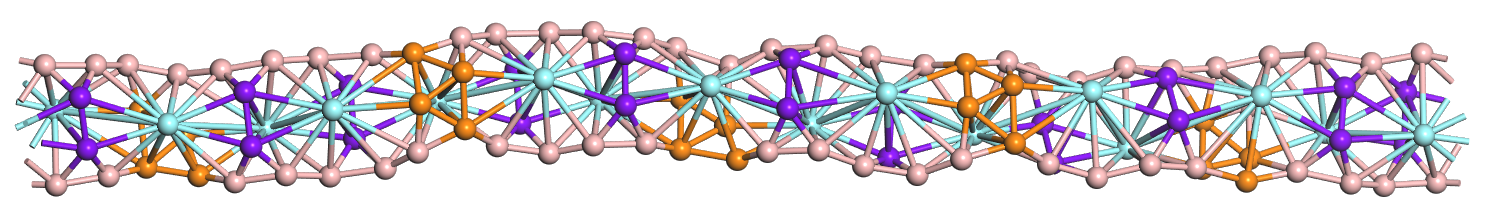
**

**FIGURE S6** The structural snapshot for 1D-Y_16_B_96_ of the FPMD simulation at 500 K at the end of 5 ps.


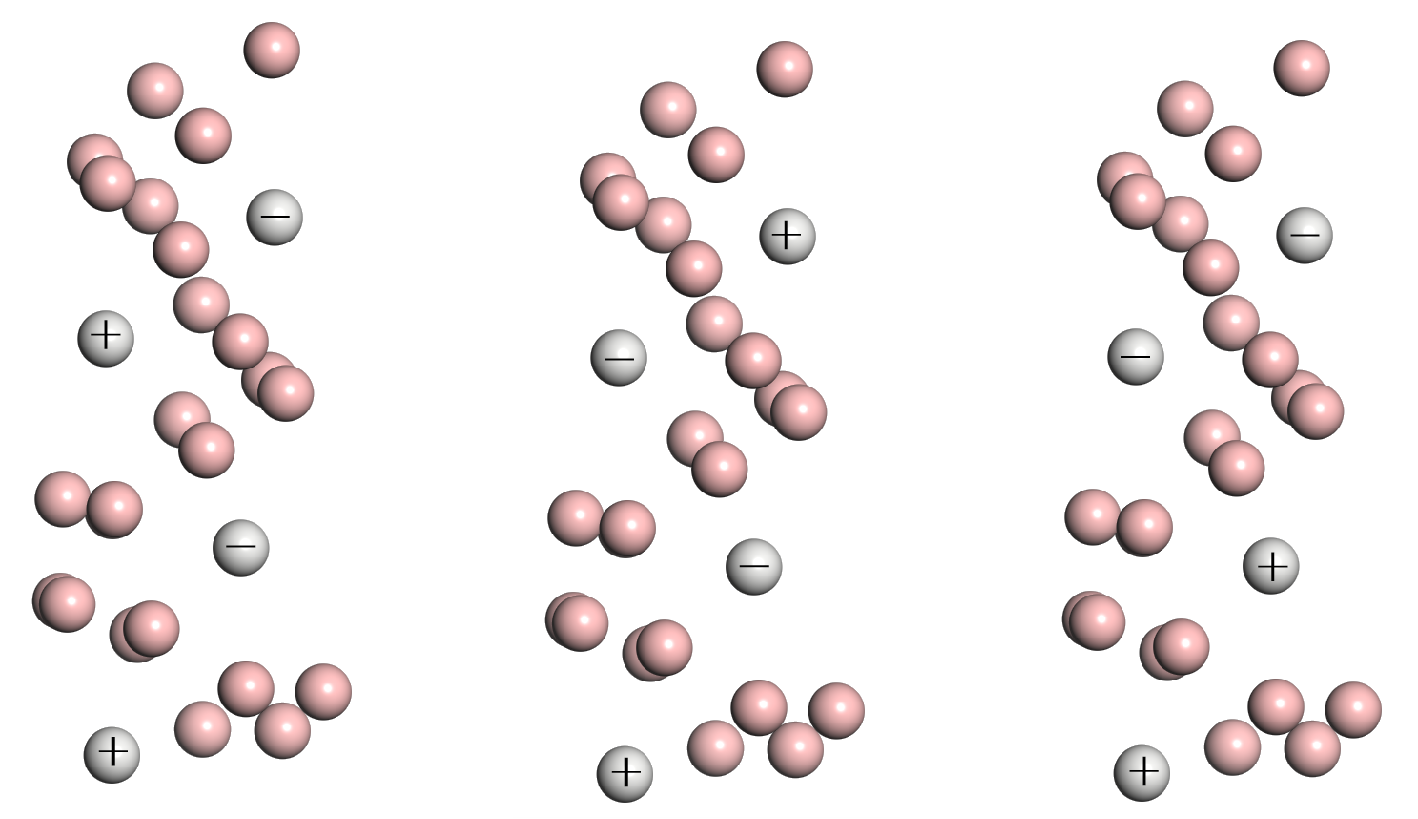


**AFM1 AFM2 AFM3**

**FIGURE S7** Magnetic configuration diagram of the designed nanowires.


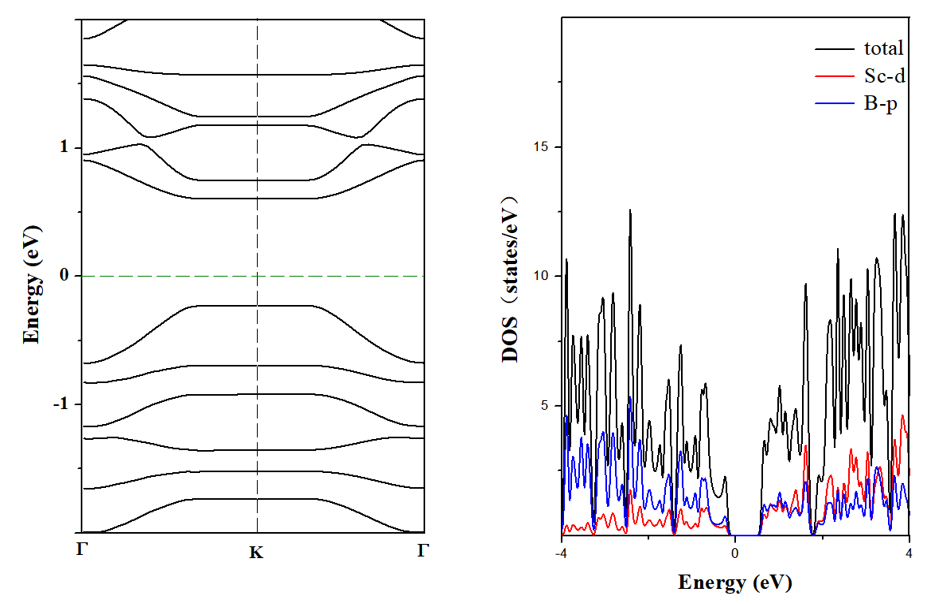


**FIGURE S8** Energy band structure and density of states of 1D-Sc_4_B_24_ nanowire predicted by HSE06 method.
